# In–situ XRD and EDS method study on the oxidation behaviour of Ni–Cu sulphide ore

**DOI:** 10.1038/s41598-017-03290-y

**Published:** 2017-06-12

**Authors:** Guangshi Li, Hongwei Cheng, Xiaolu Xiong, Xionggang Lu, Cong Xu, Changyuan Lu, Xingli Zou, Qian Xu

**Affiliations:** State Key Laboratory of Advanced Special Steel & School of Materials Science and Engineering, Shanghai University, No. 149 Yanchang Road, Shanghai, 200072 China

## Abstract

The oxidation mechanism of sulfides is the key issue during the sulphide–metallurgy process. In this study, the phase transformation and element migration were clearly demonstrated by in–situ laboratory–based X–ray diffraction (XRD) and energy–dispersive X–ray spectroscopy (EDS), respectively. The reaction sequence and a four–step oxidation mechanism were proposed and identified. The elemental distribution demonstrated that at a low temperature, the Fe atoms diffused outward and the Ni/Cu atoms migrated toward the inner core, whereas the opposite diffusion processes were observed at a higher temperature. Importantly, the unique visual presentation of the oxidation behaviour provided by the combination of in–situ XRD and EDS might be useful for optimising the process parameters to improve the Ni/Cu extraction efficiency during Ni–Cu sulphide metallurgy.

## Introduction

Since the first super–large magmatic Ni–Cu–platinum–group elements (PGE) deposit (Sudbury, Canada) was discovered in 1883, several super–large Ni–Cu–PGE deposits rich in sulphides have been mined (e.g., Jinchuan Ni–Cu–Co–PGE deposit (China), Noril’sk Ni–Cu–PGE and Pechenga Ni–Cu–PGE deposits (Russia), Voisey’s Bay Ni–Cu–Co deposit (Canada))^1^. Magmatic–sulphide deposits host ~40% of the global Ni resources and supply ~60% of the Ni in the world market. With the unceasing mining and consumption of the rich ore resources, the extensive exploitation and utilization of low–grade nickel sulphide ores are becoming important problems for the sustainability of nickel production.

**Figure 1:**
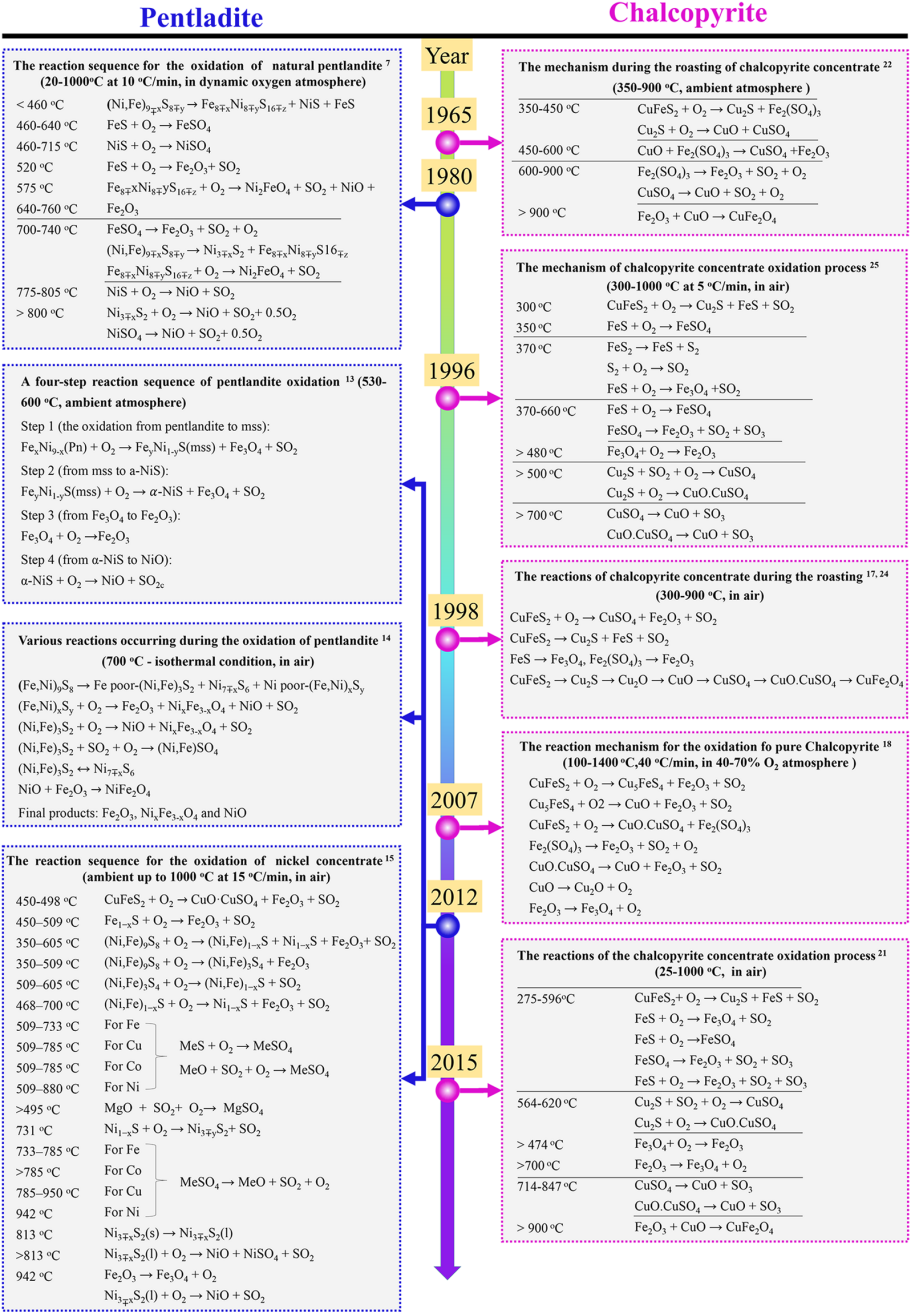
Summary of the oxidative reaction sequence for Pn and Ccp from early research.

As a traditional pyrometallurgy method, the flash smelting–converter–grinding–floatation–anode electrolysis process requires a high nickel concentrate and is tedious and complicated. From the 1960s to the 1990s, an oxidation–sulphation roasting process followed by leaching was proposed and investigated to treat low–grade Ni–Cu sulphide ores^2–6^. The oxidation behaviour of sulphide ores, which is of principal and vital importance in the sulphide–extraction metallurgy process, is complex and highly dependent on the sulphides phases in the minerals. Pentlandite (expressed as Pn) and chalcopyrite (expressed as Ccp), which are the common minerals used in pyrometallurgical nickel and copper production, respectively, have been theoretically and experimentally investigated during the oxidative–roasting process by many researchers for decades^7–16^. A detailed review of the oxidation mechanisms of Pn and Ccp is presented in Fig. 1.

Early on, the various oxidative reactions of natural Pn were deduced and summarised via thermogravimetric (TG)/mass spectroscopy (MS) analysis, differential thermal analysis (DTA), and X–ray diffraction (XRD) analysis by Dunn *et al*.^8^. Recently, researchers studied the isothermal oxidation behaviour of synthetic Pn in depth and proposed a novel stepwise phase–transformation mechanism^13, 14^. As shown in Fig. 1, pure Pn is first decomposed into intermediate sulphide (e.g., (Fe,Ni)_x_S_y_, Fe_y_Ni_1–y_S, Ni_3_S_2_, NiS) and finally oxidised into Fe_2_O_3_, NiO, and Ni_x_Fe_2+x_O_4_. For the past few years, researchers have tended to investigate the oxidation behaviour of Pn taken nickel concentrate as a raw material^15, 17–19^.

As the most exploited mineral for the extraction metallurgy of copper, Ccp has been extensively studied theoretically and experimentally^7, 9–12, 16, 20–23^. Prasad *et al*.^17^ suggested that it was extremely important and necessary for copper extraction to concentrate on the oxidative–roasting procedure of Ccp. Various reactions occurring in the oxidation of Ccp were summarised in early studies and are briefly presented in Fig. 1 ^7, 13–15, 17, 18, 21, 22, 24, 25^. As shown in this figure, the first stage is the oxidation of Ccp into Cu_2_S (or Cu_5_FeS_4_) and FeS. This is followed by the formation of metal sulphates and finally the decomposition of the sulphates of iron and copper into oxides (e.g., CuO.CuSO_4_, CuO, Fe_2_O_3_, Cu_*x*_Fe_3−*x*_O_4−*y*_). A. Baba *et al.* ^23^ conducted a detailed review of the role of Ccp in the pyrometallurgical and hydrometallurgical processes of copper, and suggested that low–grade complex sulphide ores have attracted attention because of the recent decline in deposits of high–grade ores.

Another interesting and important problem that occurs during the oxidative roasting of polymetallic sulphides is the elemental distribution of the ore grains. Many researchers^13–15, 17, 21, 22, 26^ have investigated the preferential oxidation of Fe and S during the roasting process using energy–dispersive X–ray spectroscopy (EDS), reporting that the Ni or Cu tends to diffuse into the centre while Fe migrates outward to the oxide rim layer.

Given the aforementioned findings, the oxidation mechanism of the sulphide mineral, which is a long– and well–studied problem, has been investigated in detail using traditional analysis and characterization methods, such as TG, DTA, MS, XRD and EDS. Because of the complexity of the Ni–Cu sulphide ore and the weakness of traditional characterization methods, some of the oxidation mechanisms proposed by early researchers should be further examined. However, thorough studies on the oxidative mechanism of polymetallic sulphide minerals have rarely been reported. The in–situ XRD method, which has recently been used to investigate the mechanism of the metallurgical process, is growing in popularity^27–34^. In this study, the oxidation behaviour of a Ni–Cu sulphide ore was investigated in detail during the roasting process using in–situ laboratory–based XRD and EDS in tandem.

## Results and Discussion

### Phase evolution during oxidation process

**Figure 2:**
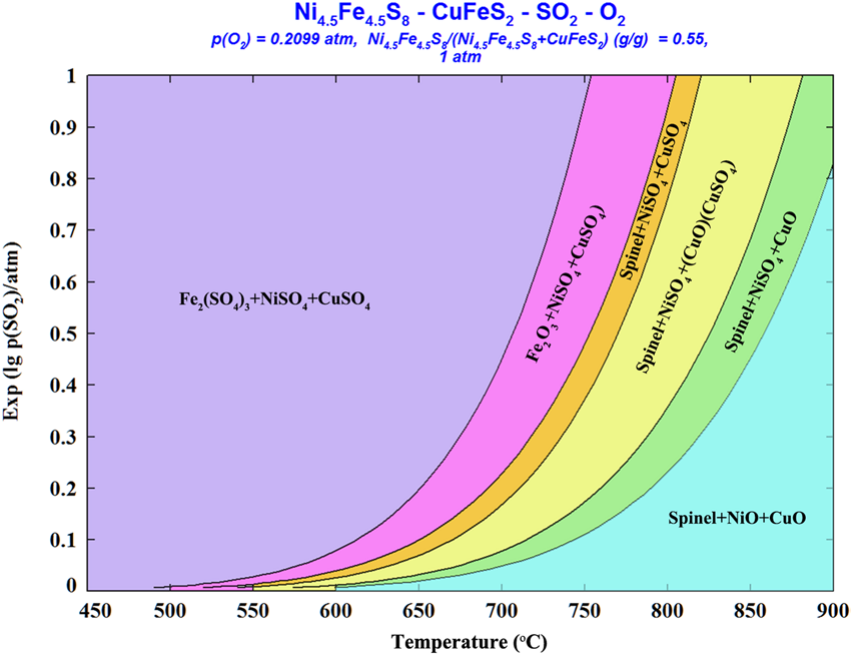
Predominance area diagram for the Ni_4.5_Fe_4.5_S_8_–CuFeS_2_–SO_2_–O_2_ system in the temperature range of 450 to 900 °C in air (p(O_2_) = 0.21 atm).

To investigate the thermodynamics of the Ni–Cu sulphide mineral system, a phase–stability diagram for the Ni_4.5_Fe_4.5_S_8_–CuFeS_2_–SO_2_–O_2_ system at 450 to 900 °C in air (p(O_2_) = 0.21 atm) was developed using the Factsage software package^35^. As shown in Fig. 2, at the lower temperatures, the main stable phases were metal sulphates (Fe_2_(SO_4_)_3_, CuSO_4_, and NiSO_4_) together with Fe_2_O_3_. As the temperature increases, the predominance areas shift toward the formation of oxides.

The TG and differential TG (DTG) curves for different heating rates in Fig. S2a and b are similar in shape, and the peaks shift to a higher temperature as the heating rate increases. The TG cures in Fig. S2a indicate that there was a gradual mass increase in the range of 400 to 550 °C and that the mass tended to decrease around 550 °C. Correspondingly, Fig. S2b shows that the formation of metal sulphates might be responsible for the two peaks (peak 1 and 2) that occurred during the mass–increasing process, whereas the other four peaks (peaks 3, 4, 5, and 6) might result from the direct oxidation of sulphides into oxides and the decomposition of the formed sulphates. Fig. S2c shows that the amount of sulphates increased at different rates as the temperature increased; reached maximums of approximately 550, 600, and 700 °C for Fe_2_(SO_4_)_3_, CuSO_4_, and NiSO_4_, respectively; and finally decomposed at higher temperatures.

**Figure 3:**
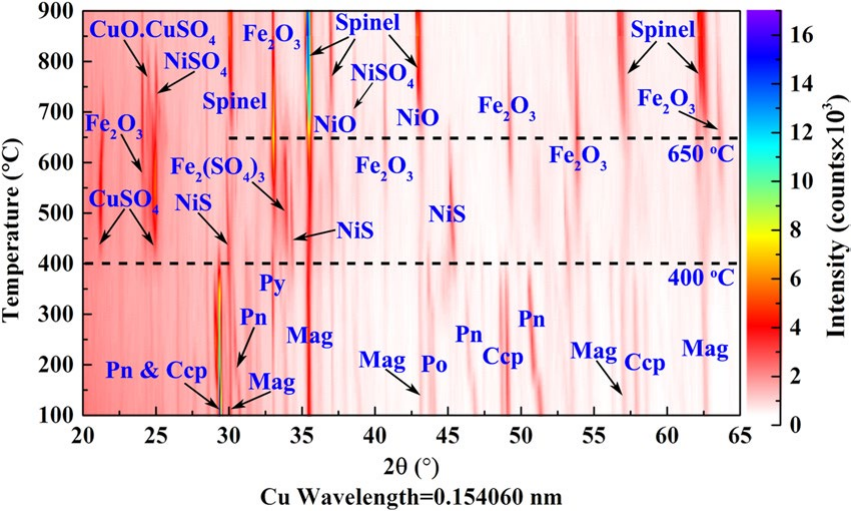
In–situ laboratory–based XRD data collected for the Ni–Cu sulphide ore sample at different temperatures (every 50 °C from 100 to 900 °C, with a heating rate of 5 °C/min) under the isothermal condition during the oxidative–roasting process.

In–situ laboratory–based XRD analysis was performed to explore the phase transformation of the Ni–Cu sulphide ore during the oxidative–roasting process. Fig. 3 plots the accumulated in–situ XRD data collected during the roasting process. And weight fractions of the phases present at each temperature are shown in Fig. S3. At “low” temperatures (here defined as <300 °C), the peak intensity of the sulphides (Pn, Ccp, and pyrrhotite (Fe_7_S_8_)) slowly decreased, but as the temperature increased, the peak intensity of pyrite (FeS_2_) increased, which indicates that Pn, Ccp, and pyrrhotite slowly decomposed^36^. When the temperature reached 350 °C or higher, the sulphides oxidised rapidly and disappeared round 400, 450 and 500 °C for Pn, Pyrrhotite and Ccp respectively, and the initial oxidative products were metal sulphates (Fe_2_(SO_4_)_3_, CuSO_4_) and NiS. In the range of 350 to 650 °C, the peak intensity of Fe_2_O_3_ gradually increased, reaching its maximum at 650 °C. As the temperature increased from 500 to 700 °C, the NiS phase gradually decreased and was eventually eliminated; however, the spinel and NiO phases exhibited a different behaviour. As the temperature increased from 650 to 900 °C, the spinel and NiO phases increased, while the Fe_2_O_3_ phase tended to decrease. As shown in Figs 3 and S3, the generation and transformation processes differed significantly among the sulphate phases. For example, the Fe_2_(SO_4_)_3_ phase appeared at 400 °C and disappeared at ~650 °C (Fig. S3c); however, the appearance and increase of the NiSO_4_ phase occurred at 550–700 °C, and the NiSO_4_ decomposed and disappeared at 850 °C (Fig. S3a). The CuSO_4_ phase was generated together with the Fe_2_(SO_4_)_3_ phase at 400 °C and then gradually disappeared with the appearance and increase of the CuO.CuSO_4_ phase at 700–800 °C. Finally, the CuO.CuSO_4_ phase disappeared at 850 °C (Fig. S3b).

**Figure 4:**
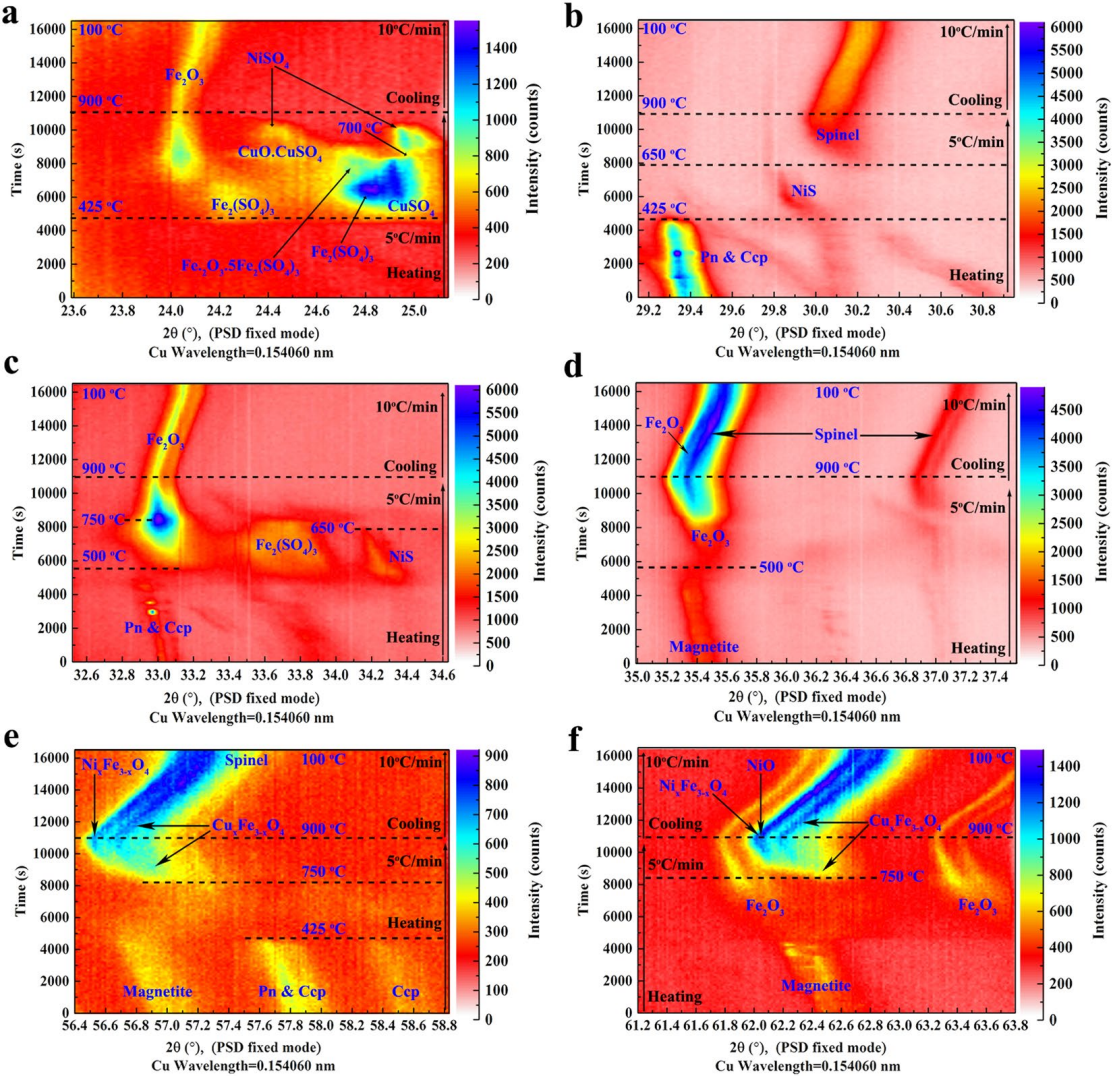
Series of characteristic peaks (2θ angle approximately 21°, 28°, 32°, 35°, 56°, and 61° for (**a**–**f** respectively) obtained with a small angle range (~2° in 2θ) using in–situ laboratory–based XRD with the PSD fixed model during the oxidative–roasting process.

For a better understanding of the oxidation behaviour of the Ni–Cu sulphide ore, a series of characteristic–peak ranges were measured using the in–situ laboratory–based XRD with the position–sensitive detector (PSD) fixed model. According to the patterns shown in Fig. 4, the oxidative processes of the Ni–Cu sulphide ore are discussed in detail as follows:

Initial oxidative stage: At the beginning, the sulphide–mineral sample hardly changed as the temperature increased. However, there may have been subtle variations in the peak intensities for Pn and Ccp, as shown in Fig. 4b and c, which indicated the slow decomposition of the sulphides^8^. Then, both the Pn and Ccp disappeared rapidly at ~425 °C, as shown in Fig. 4b,c and e. The Mag was gradually oxidised into Fe_2_O_3_ above 425 °C, as shown in Fig. 4d,e and f.

Sulphation stage: As shown in Figs 4a,c and S3, the sulphates (Fe_2_(SO_4_)_3_ and CuSO_4_) were formed almost instantaneously around 425 °C and increased gradually with the increase of the oxidation temperature. Figure 4a shows that the Fe_2_(SO_4_)_3_ phases gradually transformed into iron oxide (Fe_2_O_3_) and iron oxide sulphate (Fe_2_O_3_.5Fe_2_(SO_4_)_3_) in the temperature range of 500 to 700 °C. Then, the iron oxide sulphate (Fe_2_O_3_.5Fe_2_(SO_4_)_3_) decomposed into Fe_2_O_3_ and disappeared at ~750 °C.

As shown in Fig. 4a, CuSO_4_ was rapidly generated around 425 °C and remained stable below ~600 °C. CuO.CuSO_4_, which was detected in the range of 650 to 750 °C, was the decomposition product of CuSO_4_ (Figure S3b). However, as shown in Fig. 4b and c, the Pn was first transformed into the NiS phase in the temperature range of 425 to 500 °C, and then the NiS phase gradually increased with the temperature, disappearing at ~650 °C. As shown in Fig. 4a, NiSO_4_ was formed in a higher temperature range—700 to 800 °C—which is consistent with the range shown in Fig. S3a (600 to 800 °C).

Spinel–formation stage: As demonstrated in Fig. 4c, the iron oxide (Fe_2_O_3_) increased rapidly around 500 °C and reached its maximum value when the temperature increased to ~750 °C. In addition, as shown in Fig. 4a and c, the remarkable changes of the Fe_2_O_3_ diffraction peak profile occurred during the heating and cooling processes, which might be the results of the growth of Fe_2_O_3_ crystal. As shown in Fig. S4, the [104] crystal size of Fe_2_O_3_ firstly increased at the heating stage, remained constant at the temperature range from 600 °C to 700 °C, and increased at temperature up to 800 °C, then remained unchanged until the cooling stage. The spinel phase was initially generated around 750 °C, as shown in Fig. 4b,d,e and f. Then, the spinel phase gradually increased with the temperature. In contrast, the Fe_2_O_3_ was reduced as the temperature increased. As shown in Fig. 4e and f, the diffraction patterns of the Ni–spinel and Cu–spinel phases were separated and revealed clearly, whereas the diffraction peaks of the NiO and Ni–spinel phases exhibited serious overlap. The Cu–spinel was formed with priority, and then the Ni–spinel and NiO peaks were detected.

Cooling stage: The phase components of the sample remained constant in the cooling stage, as shown in Fig. 4. The diffraction peaks significantly shifted to a large angle because of the lattice contraction during the cooling process. Conversely, the lattice expansion during the roasting caused the peaks to shift to a small angle.

### Elemental migration and distribution

**Figure 5:**
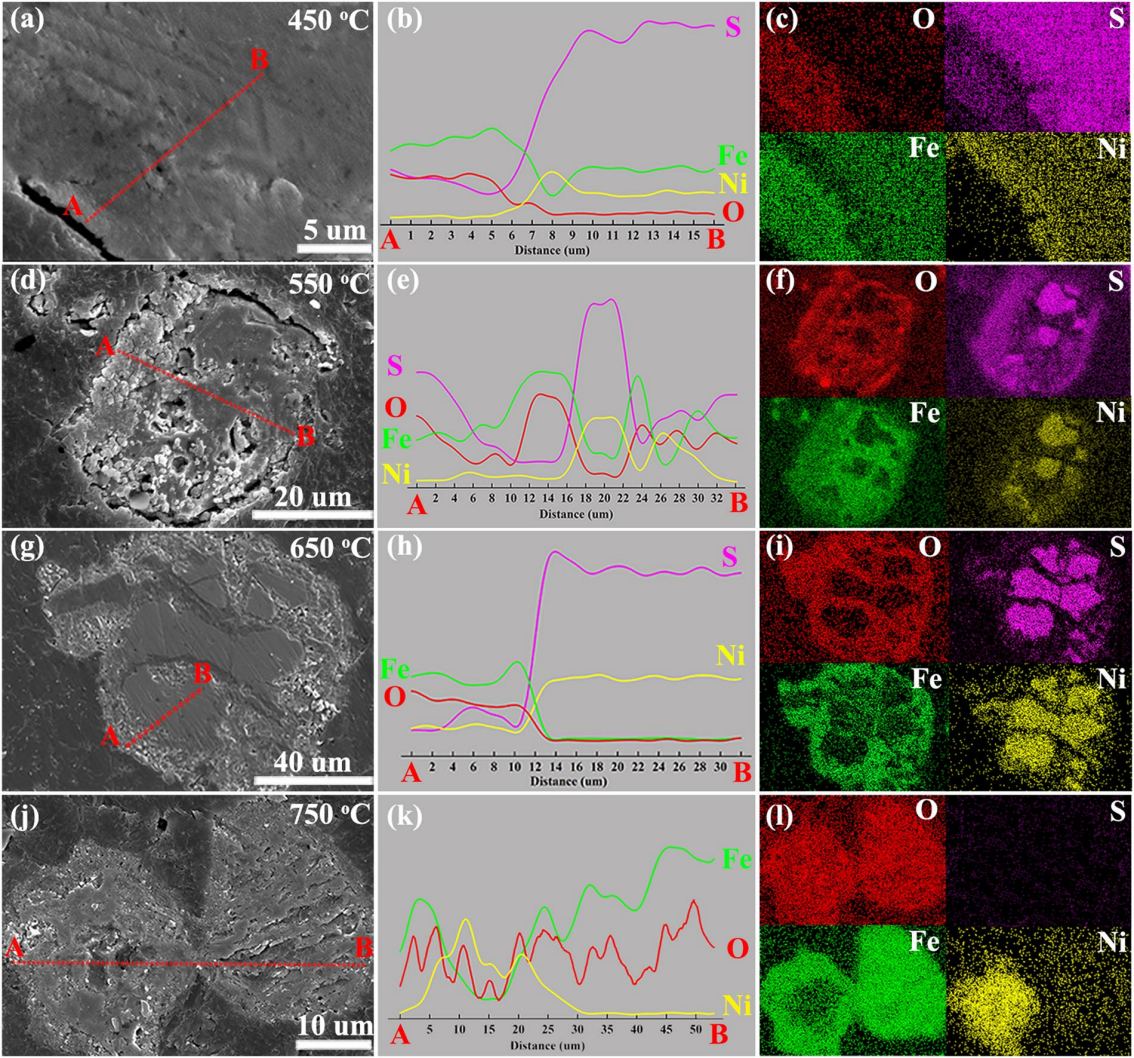
SEM, EDS line–scanning and EDS–mapping results for elements (O, S, Fe, and Ni) over the cross section of Pn particles after the roasting at 450 °C **(a**–**c**), 550 °C (**d**–**f**), 650 °C (**g**–**i**), and 750 °C (**j**–**l**) in air.

**Figure 6:**
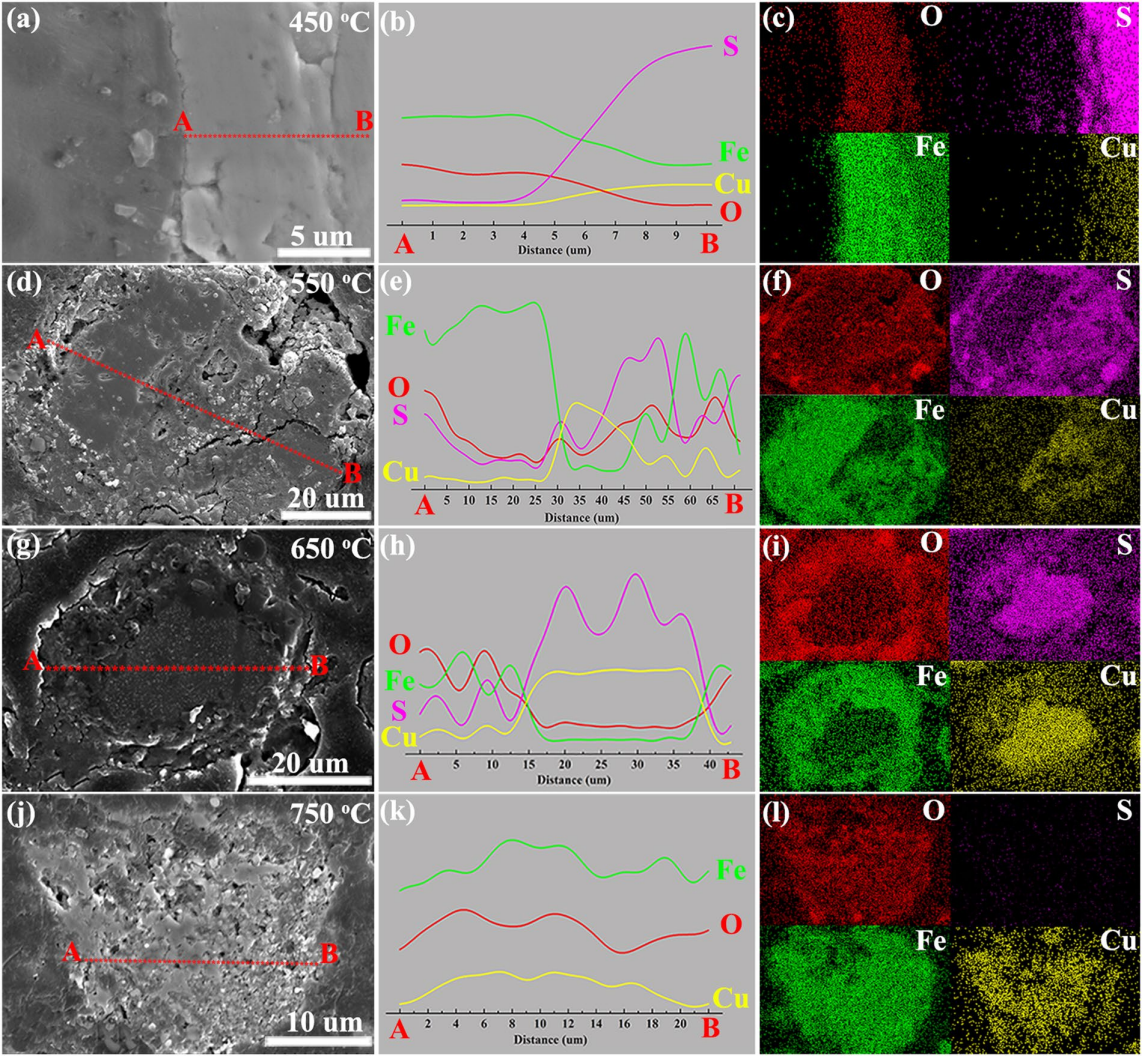
SEM, EDS–line–scanning, and EDS–mapping results for elements (O, S, Fe, and Cu) over the cross section of Ccp particles after the roasting at 450 °C (**a**–**c**), 550 °C (**d**–**f**), 650 °C (**g**–**i**), and 750 °C (**j**–**l**) in air.

The elemental distribution of the cross–section of the particles was studied by EDS, as discussed in this section. The SEM and EDS analysis of Pn and Ccp grains at different temperatures are shown in Figs S5 and S6, respectively. The element migration in the Pn and Ccp grains was similar in the initial oxidative stage (~450 °C). As shown in Figs 5b and 6b, the Fe atoms in Pn and Ccp migrated to the surface and preferentially oxidised into a dense, thin oxide layer (4–5 µm), whereas the Ni and Cu tended to migrate to the centre of the grains in the earlier oxidation stage. Fig. 5c shows that there were three layers of Pn grains at this stage. According to the in–situ XRD (Figs 3 and 4) and EDS spot (Fig. S5a) analyses, these three layers were the Fe_2_(SO_4_)_3_ outer layer, the intermediate thin band of the NiS phase, and the inner core of Pn or (Ni,Fe)_9_S_8–*x*_ (Pn’). However, for the Ccp particles (Fig. 6c and Fig. S6a), the outer side was an Fe_2_O_3_ dense layer, while the inner side was Ccp or CuFeS_2–*y*_ (Ccp’).

As the temperature increased to 550 °C (Figs 5e, 6e and Figs S5b, S6b), the oxidised gas product (SO_2_) diffusing outward collided with the O_2_ undergoing inward diffusion at the rim of the Fe_2_O_3_ layer. As shown in Figs 5f and 6f, the Fe_2_(SO_4_)_3_ outer rims were then formed at both the Pn and Ccp grains. Simultaneously, the inward diffusion of O_2_ resulted in Ni– and Cu–enriched cores of Pn and Ccp, respectively. The Cu–enriched core further sulphated into CuSO_4_, whereas the Ni–S enriched core remained stable at 550 °C. This phenomenon is consistent with the sulphate–leaching experiments (Fig. S2a) and the in–situ XRD characterizations (Figs 3 and 4). The aforementioned results suggest that the sulphation of Ccp was preferential over that of Pn. As shown in Figs 5h and 6h, the distribution of S at the outer layer decreased or disappeared, mainly owing to the decomposition of the outer Fe_2_(SO_4_)_3_ layers into Fe_2_O_3_ at 650 °C (Figs S5c and S6c). A clearer and more realistic view of this is provided by comparison with Fig. 5f and i (Fig. 6f and i for Ccp grain).

As shown in Fig. 5k and l, the NiS inner core was oxidised into NiO and Ni_*x*_Fe_3−*x*_O_4−*y*_ at 750 °C. There were three layers (NiO–Ni_*x*_Fe_3−*x*_O_4−*y*_–Fe_2_O_3_, from inner to outside) in the Pn grain (Fig. S5d). Surprisingly, however, the sulphation of Ni is not been observed in Fig. 5, possibly because a small amount of NiSO_4_ formed under this continuous–heating condition and the grains were not selected randomly in this work. For the Ccp grains (Fig. 6k and l), there may have been a Cu_*x*_Fe_3−*x*_O_4−*y*_ inner layer with a Fe_2_O_3_ thin surface after the roasting at 750 °C (Fig. S6d). Another noteworthy phenomenon is that the Ni tended to diffuse outward gradually, as observed by comparing the line–scanning results in Fig. 5h and k (Fig. 5i and l for mapping). Moreover, as shown in Fig. 6k and l, diffuse distributions of Cu, Fe, and O appeared, indicating that the Cu atoms diffused outward more rapidly than the Ni atoms.

### Oxidation mechanism of Ni–Cu sulphide ore

**Figure 7:**
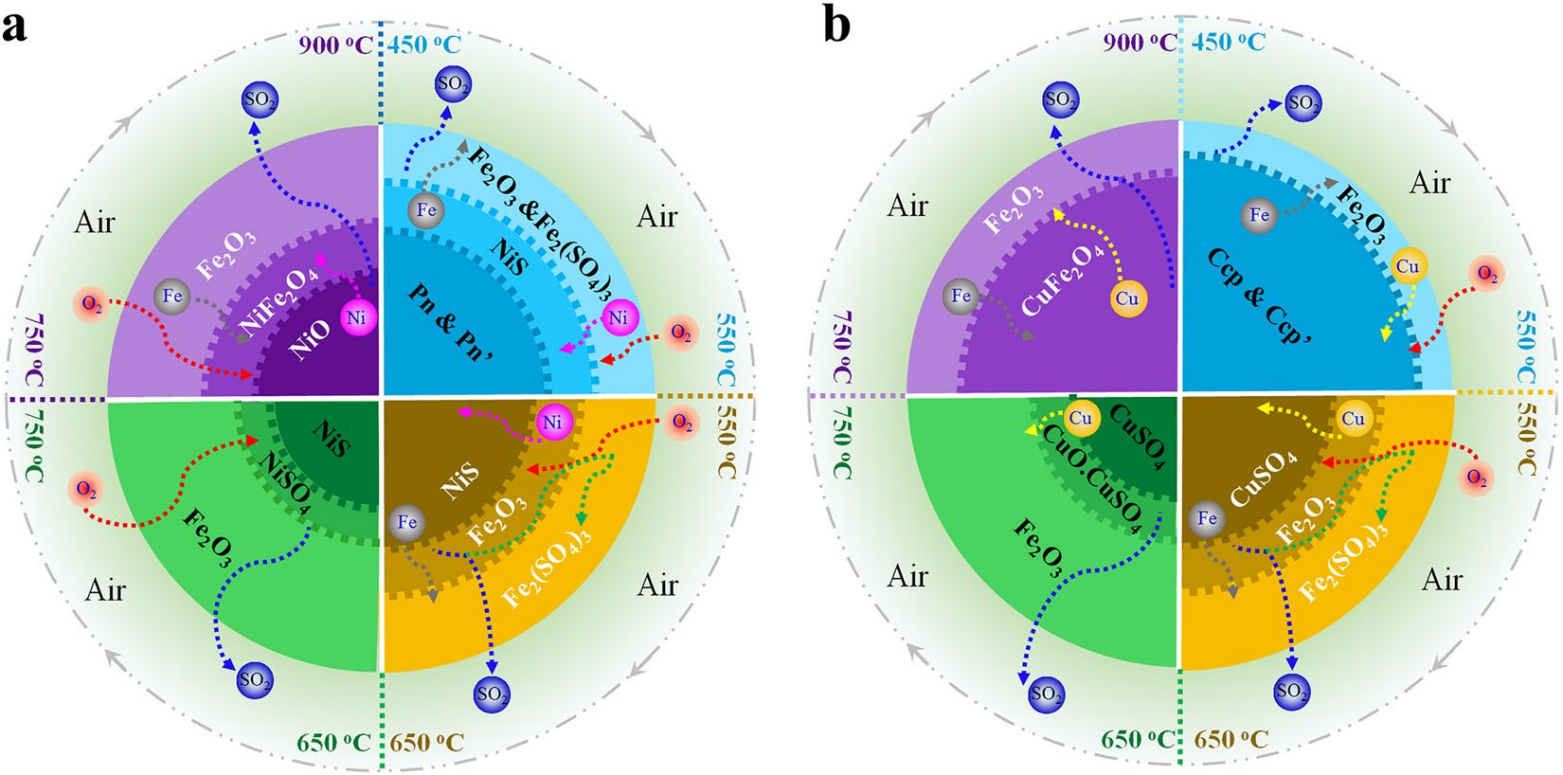
Schematic of the oxidative–roasting mechanisms of Pn (**a**) and Ccp (**b**) in the Ni–Cu sulphide ore. Four temperature ranges were defined, and a four–stage reaction sequence occurred.

Considering the aforementioned results, the reaction sequence of the Ni–Cu sulphide ore during the roasting in air was deduced, as summarised in Table 1. The oxidation mechanism of Pn and Ccp is proposed as follows and shown in Fig. 7. In the temperature range of 350 to 450 °C, the surface–absorbed O_2_ first contacted and reacted with the mineral grains, and a thin Fe_2_O_3_ layer was formed at this stage. Fe and S were preferentially oxidized over Ni and Cu, which was clearly observed and well–investigated. Consequently, the Fe atoms diffused to the surface, while the Ni and Cu atoms migrated into the inner core. Although many authors have observed and investigated this phenomenon, no reasonable theory has been proposed to explain the internal connection between the preferential oxidation of Fe and the opposite diffusion directions of Fe and Ni (or Cu)^13–15, 17, 18, 22^. Recently, researchers used the ab–initio density functional theory to investigate and explain the preferential oxidation of Fe at the Pn and Ccp surface^37^. And, based on the characterization presented in the present study, this preferential oxidation may have resulted from the chemical–affinity of metals (Fe, Ni and Cu) for oxygen and sulphur^38^. While the Fe was preferentially oxidized into Fe–O phase on the surface of Pn or Ccp crystal structure, the concentration of Fe–S declined sharply, resulting in the outward migration of Fe atoms. Then the Ni/Cu atoms diffused into the inner core and formed Ni–S or Cu–S phase.

**Table 1:** Summary of the reaction sequence that occurred during the oxidative roasting of Ni–Cu sulphide in air.

350–425 °C	(Ni,Fe)_9_S_8_ (Pn) → (Ni,Fe)_9−*y*_S_8–x_ (Pn’) + FeS + NiS
	CuFeS_2_ (Ccp) → CuFe_1−*x*_S_2–x_ (Ccp’) + xFeS
	Fe_7_S_8_ (Pyrrhotite) → 6FeS + FeS_2_
425–500 °C	Pn’ + O_2_ → NiS + Fe_2_(SO_4_)_3_
	2FeS_2_ + 7O_2_ → Fe_2_(SO_4_)_3_ + SO_2_
	4Fe_3_O_4_ (Magnetite) + O_2_ → 6Fe_2_O_3_
	2Fe_3_O_4_ + 9SO_2_ + 5O_2_ → 3Fe_2_(SO_4_)_3_
425–650 °C	2CuFeS_2–y_ (Ccp’) + (9-2y)O_2_ + (1 + 2y)SO_2_→ 2CuSO_4_ + Fe_2_(SO_4_)_3_
500–650 °C	2NiS + 3O_2_ → 2NiO + 2SO_2_
	6Fe_2_(SO_4_)_3_ → Fe_2_O_3_.5Fe_2_(SO_4_)_3_ + 3SO_3_ → 6Fe_2_O_3_ + 12SO_2_
650–750 °C	NiS + 2O_2_ → NiSO_4_
	2NiO + 2SO_2_ + O_2_ → 2NiSO_4_
650–800 °C	2CuSO_4_→ CuO.CuSO_4_ + SO_3_
750–900 °C	NiSO_4_ + Fe_2_O_3_ → NiFe_2_O_4_ + SO_3_
	NiO + Fe_2_O_3_ → NiFe_2_O_4_
	CuO. CuSO_4_ + 2Fe_2_O_3_ → 2CuFe_2_O_4_ + SO_3_

As the temperature increased from 450 to 550 °C, Fe and S were continuously preferentially oxidized over Ni and Cu, but the Fe_2_O_3_ remained at the interlayer between the outer Fe_2_(SO_4_)_3_ layer and the inner core (NiS for Pn grain, CuSO_4_ for Ccp grain) at this stage. A large amount of SO_2_ gas, which was produced by the oxidation of Fe in the interlayer, diffused outward, possibly reacted with O_2_ to form SO_3_, and then transformed the previously formed Fe_2_O_3_ layer into the outer Fe_2_(SO_4_)_3_ layer. At this stage, the domain reaction was the sulphation of Fe and Cu, and the NiS in the inner core remained stable.

The sulphation of Ni occurred at a higher temperature (approximately 650–750 °C), and a small amount of NiSO_4_ was formed at the internal layer between the NiS core and the Fe_2_O_3_ outer rim at this stage, owing to the small amount of SO_2_ concentrate produced by the decomposition of Fe_2_(SO_4_)_3_ and CuSO_4_. A small part of the CuSO_4_ decomposed and formed a CuO.CuSO_4_ rim, yet most of the CuSO_4_ remained stable in the inner core. Hence, this stage was suitable for the sulphation roasting of nickel concentrate, which simultaneously leached Ni and Cu in water and separated Fe from the Ni–Cu sulphide mineral.

The final stage involved the decomposition of CuO.CuSO_4_, CuSO_4_, and NiSO_4_ and the formation of the spinel phase (M_*x*_Fe_3−*x*_O_4−*y*_, M = Fe, Co, Ni, Cu, Mg). The Ni and Cu tended to react with the Fe_2_O_3_ to form a stable spinel phase. The high chemical stability of the spinel phase may lead to the formation of chemical potential at the reactive interface, then may have caused the outward diffusion of Cu/Ni and the inward diffusion of Fe at a higher temperature^39–43^. Moreover, the Cu_*x*_Fe_3−*x*_O_4−*y*_ was diffusely distributed in the grain, while the Ni_*x*_Fe_3−*x*_O_4−*y*_ was just an internal rim with the inner core of NiO. And it may be due to the difference in cation diffusion rates between Ni and Cu during the roasting process.

In conclusion, the oxidation behaviour of a Ni–Cu sulphide ore, which is closely related to the sulphide–extraction metallurgy process, was thoroughly investigated by in–situ laboratory–based XRD and EDS. A four–step oxidation mechanism, including the preferential oxidation of Fe and S, metal sulphation, the decomposition of sulphates, and the formation of a spinel phase, was proposed according to phase–diagram analysis, TG–DTG analysis, the sulphate–leaching process, and in–situ XRD patterns. The reaction sequence that occurred during the oxidation of the Ni–Cu sulphide ore was summarised. In addition, the EDS elemental distribution demonstrated that the Fe atoms diffused outward and the Ni/Cu atoms migrated toward the inner core during the first three stages, whereas these diffusion processes were opposite at the final stage, possibly because of chemical–affinity of metals (Fe, Ni and Cu) for oxygen and sulphur and the high chemical stability of the spinel phase, respectively. A unique visual characterization of the oxidation mechanism in the Ni–Cu sulphide ore roasting process was performed by a combination of in–situ XRD and EDS. This mechanism may be conducive to an alternative metallurgy process for the Ni–Cu sulphide ore.

## Methods

### Sample preparation

A Ni–Cu sulphide ore sample, which was a product of flotation and originated from Jinchuan Group Ltd., China, was investigated. The powder sample was dried at 100 °C for 24 h and passed over vibrating sieves to obtain an extremely fine powder with a diameter of less than 74 um. XRD analysis indicated that the sample was composed of Pn, magnetite, Ccp, pyrrhotite, lizardite, pyrite, and quartz. XRD quantitative phase analysis (QPA) was performed using the Rietveld refinement method with the TOPAS software^44–47^. The Rietveld refinement of the diffraction pattern resulted in good–quality fits with a goodness of fit (Gof) of nearly 2, as shown in Fig. S1. The results of the XRD quantitative phase analysis of the Ni–Cu sulphide ore sample are presented in Table 2. As shown in Table 3, the content of the major metallic elements (Fe, Ni, Cu) in the sample was determined by X–ray fluorescence (XRF), inductively coupled plasma atomic emission spectroscopy (ICP–AES, for short), and XRD analyses.

**Table 2:** XRD quantitative phase analysis of the Ni–Cu sulphide ore sample.

Mineral	Pentlandite	Magnetite	Chalcopyrite	Pyrrhotite	Lizardite	Pyrite	Quartz
wt%	27.3	23.5	22.8	14.4	4.0	5.1	2.7
Errors%	1.7	1.8	1.5	0.9	0.5	0.5	0.4

**Table 3:** Content of the major metallic elements (Fe, Ni, Cu) in Ni–Cu sulphide ore sample, determined by XRF, ICP, and XRD.

Fe			Ni			Cu		
XRF	ICP	XRD	XRF	ICP	XRD	XRF	ICP	XRD
28.3%	34.2%	44.8%	7.7%	9.4%	8.3%	6.5%	6.6%	7.9%

### TG analysis and oxidative roasting–water leaching experiments

TG analysis was performed to evaluate the oxidation behaviour of Ni–Cu sulphide ore powder samples using a TG analyser (NETZSCH STA 449 F3 Jupiter) at different heating rates (5, 10, and 20 °C/min) in air. 2 g of the Ni–Cu sulphide ore powder sample was put into a crucible (30 mm × 70 mm) and then the crucible was placed in a muffle furnace, which was heated to a target temperature (from 100 to 900 °C in intervals of 50 °C) at a rate of 5 °C/min and maintained for 30 min. The calcines were then leached in 150 mL of water at 80 °C for 2 h to evaluate the amount of sulphate species formed during the oxidative–roasting process. After filtration, the filtrate was analysed by ICP–AES.

### In–situ XRD (full–pattern scanning and PSD fixed model)

In–situ laboratory–based XRD experiments were performed using a laboratory X–ray powder diffractometer (Bruker D8 ADVANCE, with Cu Kα1 radiation). An Anton Paar XRK 900 reactor chamber with a Macor sample holder, which was designed for the in–situ XRD investigation of solid–state and solid–gas reactions at temperatures ranging from 25 to 900 °C, was fitted to the diffractometer. The powder sample rested upon a ceramic sieve (pore size of 0.2 mm), so that reactive gas could penetrate the sample and sample holder. XRK 900 was connected to a TCU 750 temperature–control unit. The heating procedure and measuring parameters were set using the control software of the X–ray diffractometer. The in–situ XRD experiments focused on the oxidative–roasting process of the Ni–Cu sulphide ore sample under a 40 mL/min flow of air steam.

For the full–pattern scanning model of the in–situ XRD, the sample was heated from 25 to 900 °C at 5 °C/min. XRD patterns were collected at different temperatures (every 50 °C from 100 to 900 °C °C) under the isothermal condition. To ensure that the reactions approach chemical equilibrium, a delay of 30 min was set for the data collections. The patterns were recorded in the 2θ range of 20 to 65° with a step size of 0.02° and a counting time of 1 s per step. The diffraction patterns were analysed by Rietveld refinement using TOPAS software^44–47^, allowing phase abundances to be quantified.

For the PSD fixed model of the in–situ XRD, the detector was set at a fixed angle to enable the simultaneous detection of a certain 2θ range of the diffraction–pattern data. The sample was roasted from 25 to 900 °C at 5 °C/min and cooled to room temperature at −10 °C/min. A certain 2θ angle (approximately 21°, 28°, 32°, 35°, 56°, and 61°) was set as the starting angle to collect the characteristic diffraction–pattern data instantaneously throughout the heating and cooling process. The opening slit of the detector was set as 2.8° (the maximum value), allowing us to obtain diffraction patterns over the maximum 2θ range. The diffraction data were collected with a counting time of 2 min for each point, yielding temperature resolutions of 10 and 20 °C during the heating and cooling, respectively.

### EDS characterization of oxidative–roasting samples

The Ni–Cu sulphide ore sample was evenly sprinkled on four corundum crucibles, forming ultrathin layers of mineral particles at the bottom of each crucible. The four resulting samples were roasted in a muffle furnace in the ambient atmosphere from 25 °C to 450, 550, 650, and 750 °C, respectively, at a heating rate of 5 °C/min. When the given temperature was reached, the samples were removed from the furnace and immediately quenched in liquid nitrogen. The cooling oxidative–powder samples were mounted in epoxy resin. Then, the cured epoxy samples were ground with metallographic abrasive paper and polished on a diamond pad with an oil–based 0.3–µm diamond suspension as the polishing media. Finally, the polished samples were coated with Pt and then characterised using a scanning electron microscope (SU–1500, Hitachi) complemented by an energy–dispersive X–ray detector (Oxford Inca X–Max).

## Electronic supplementary material

Supplementary Information
