# Supplementary material for: In–situ XRD and EDS method study on the oxidation behaviour of Ni–Cu sulphide ore
Source: Sci Rep. 2017 Jun 12;7:3212. doi: 10.1038/s41598-017-03290-y (PMC5468282; doi:10.1038/s41598-017-03290-y)
Supplement: Supplementary file 1 — Supplementary Information [file 41598_2017_3290_MOESM1_ESM.doc]

**In–situ XRD and EDS method study on the oxidation behaviour of Ni–Cu sulphide ore**

Guangshi Li ^1^, Hongwei Cheng ^1^,*, Xiaolu Xiong ^1^, Xionggang Lu ^1^,*, Cong Xu ^1^, Changyuan Lu ^1^, Xingli Zou ^1^, Qian Xu ^1^

^1^ State Key Laboratory of Advanced Special Steel & School of Materials Science and Engineering, Shanghai University, No.149 Yanchang Road, Shanghai 200072, China


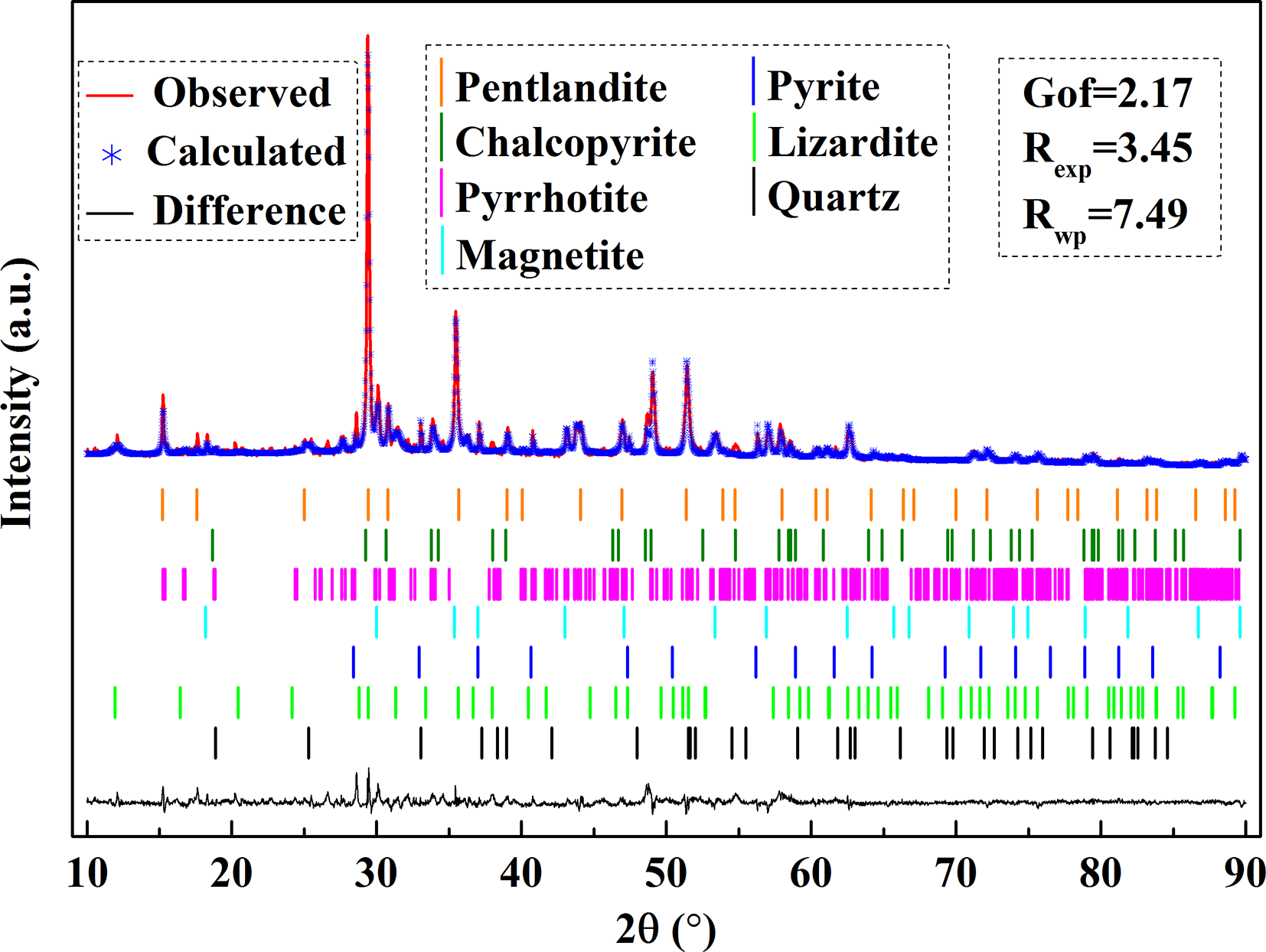


**Figure S1.** Rietveld refinement plot of the Ni–Cu sulphide ore sample: The asterisk “
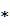
” indicates calculated data; the continuous red line indicates the observed data; the grey line shows the residual curve. The Bragg peak positions for each mineral are indicated by vertical lines. The refinement quality is indicated by the R-weighted pattern (R_wp_), R-expected (R_exp_), and Gof.


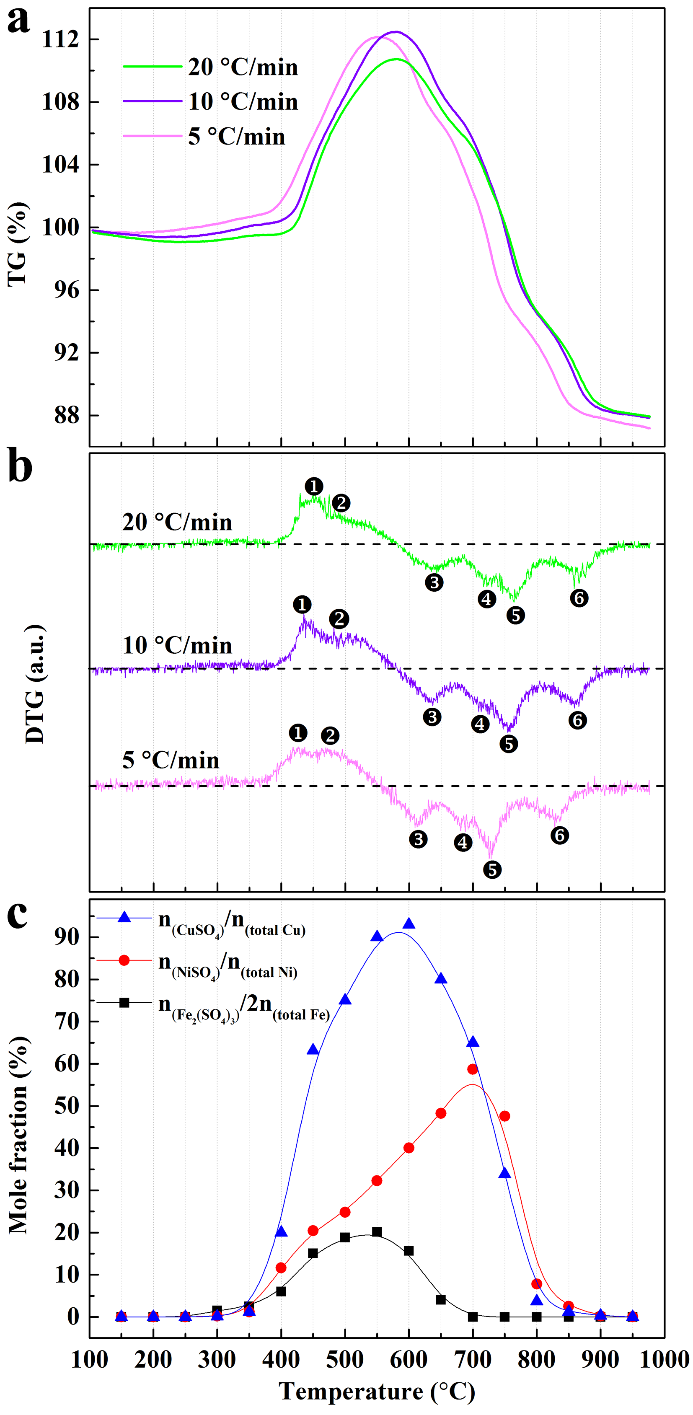


**Figure S2. a.** TG and **b.** differential TG (DTG) curves of the Ni–Cu sulphide ore at different heating rates during the oxidative roasting; **c.** amount of sulphates formed at different temperatures during the oxidative roasting.


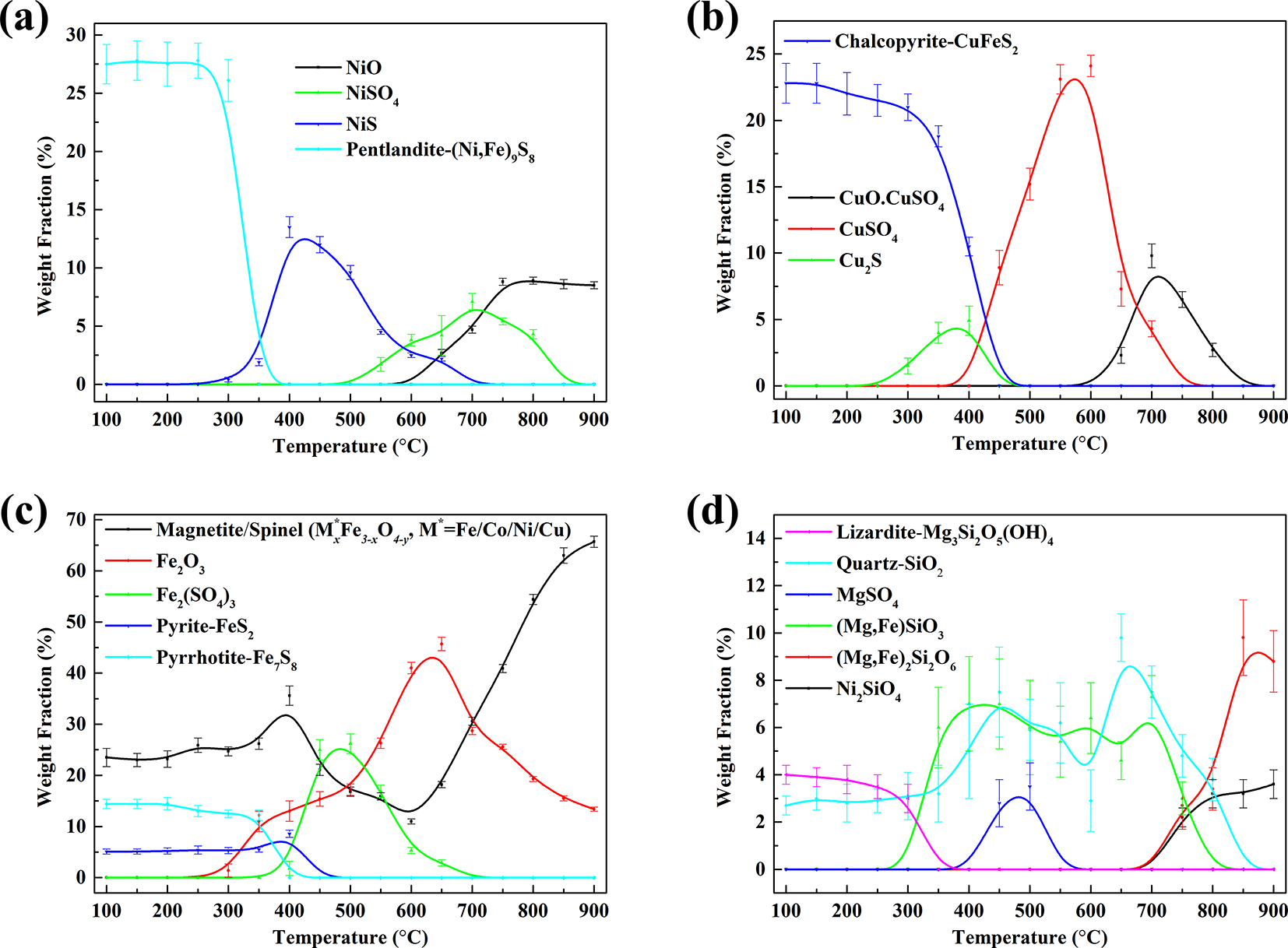


**Figure S3.** Results of QPA for the in–situ laboratory–based XRD experiment (full–pattern scanning model) data performed by the Rietveld refinement method with the TOPAS software, showing relative phase abundances as a function of temperature: phases containing Ni (a), Cu (b), Fe (c) and Mg/Si (d).


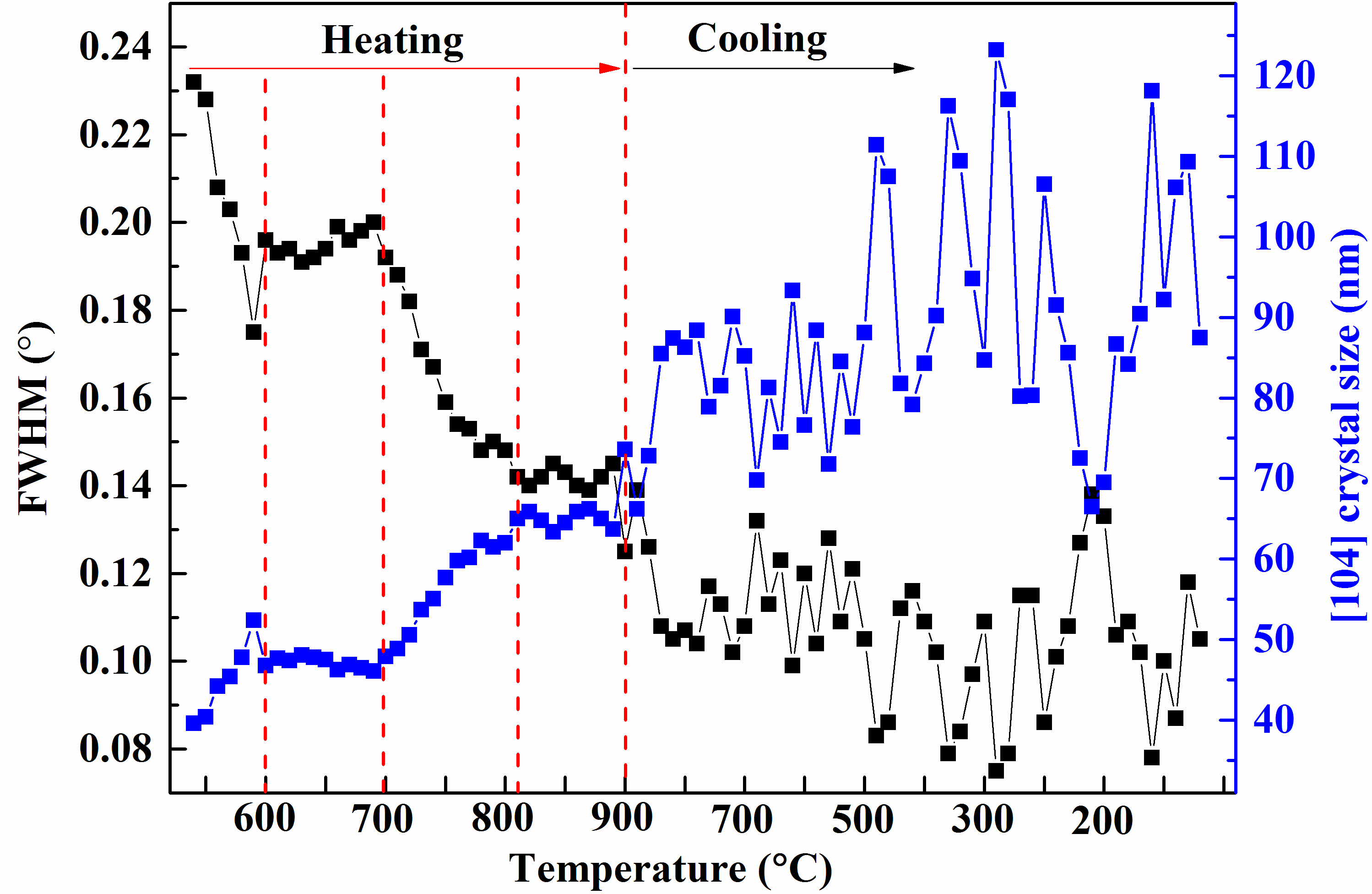


**Figure S4.** The FWHM and crystal size of (104) peak of Fe_2_O_3_ during the heating and cooling process.


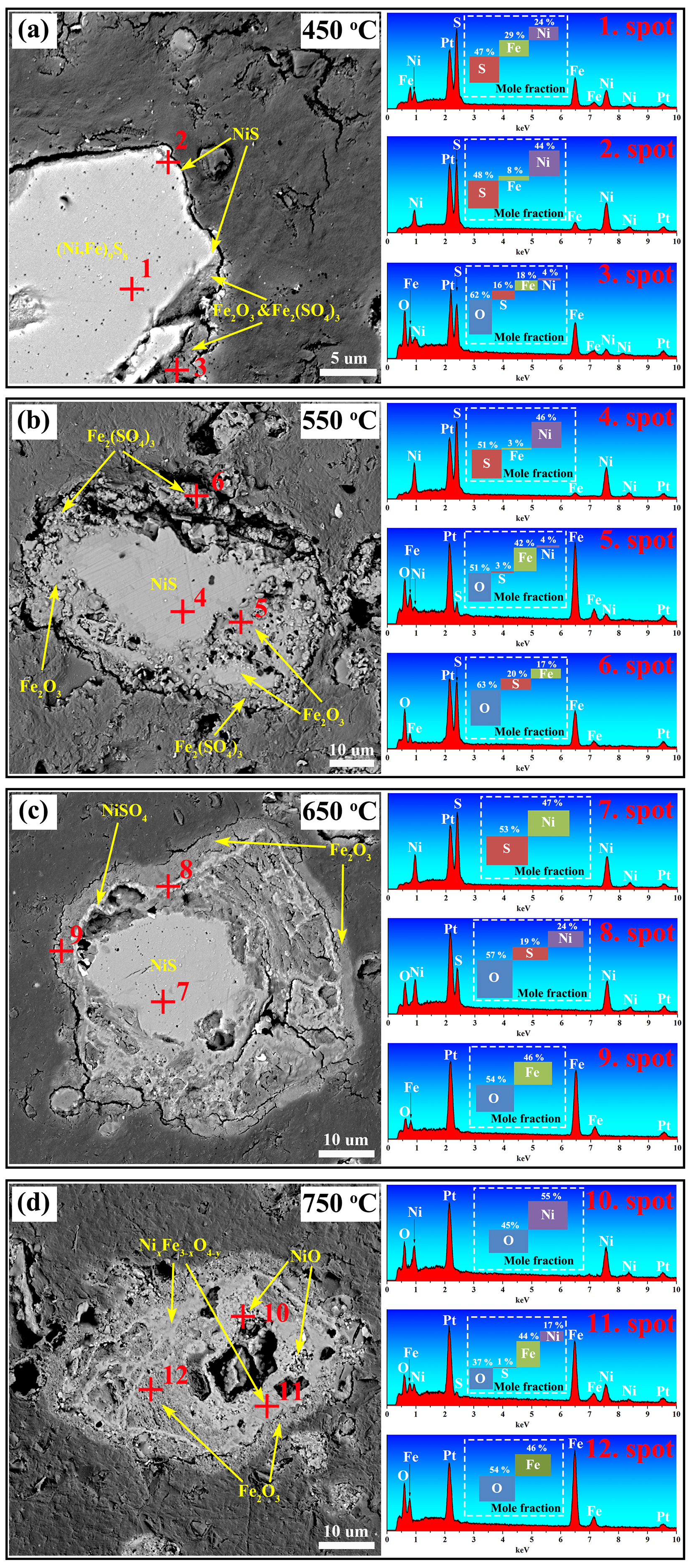


**Figure S5.** SEM micrographs (back scattered electron) and EDS spot analysis over the cross section of Pn particles after the roasting at 450 °C (a), 550 °C (b), 650 °C (c), and 750 °C (d) in air.


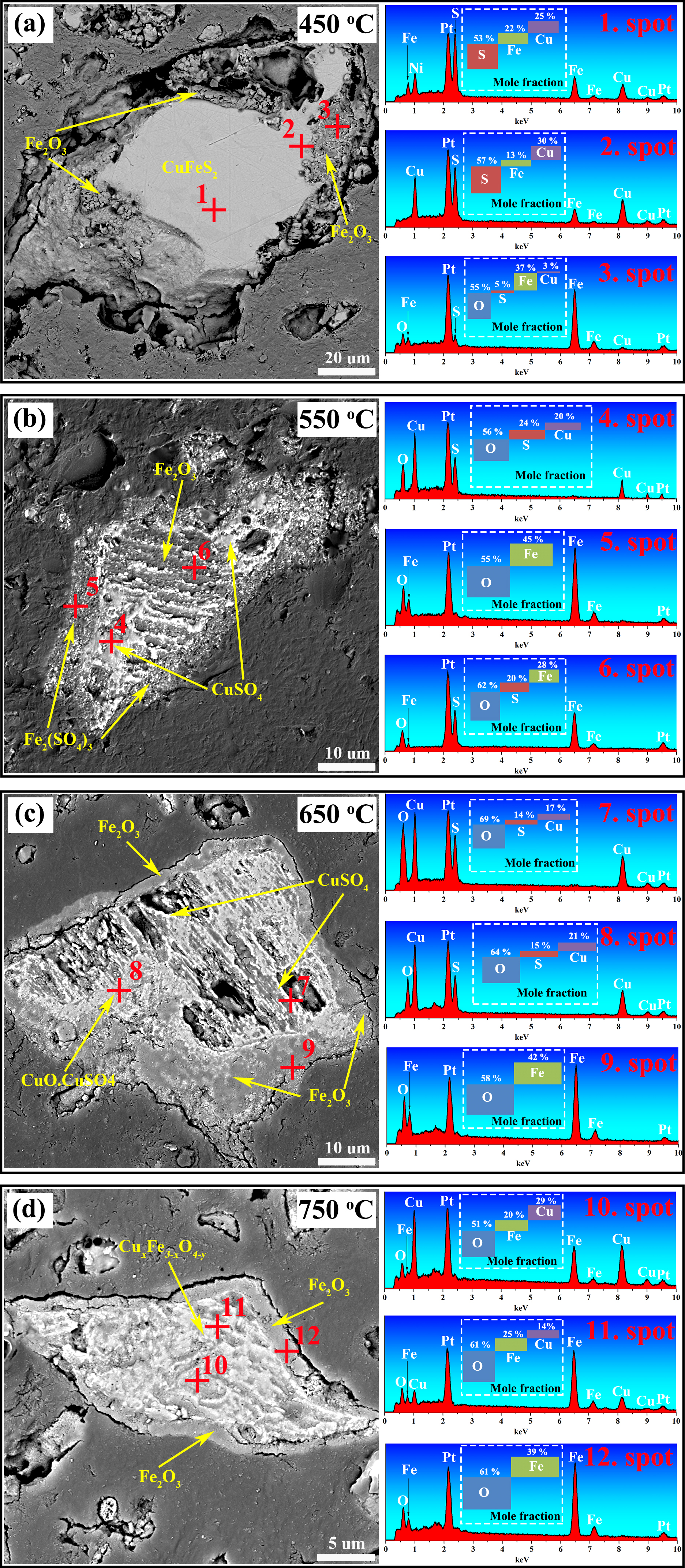


**Figure S6.** SEM micrographs (back scattered electron) and EDS spot analysis over the cross section of Ccp particles after the roasting at 450 °C (a), 550 °C (b), 650 °C (c), and 750 °C (d) in air.

**Table S1.** Phase identifications of the XRD patterns

| **Chemical formula** | **Database_code_PDF** | **Chemical formula** | **Database_code_PDF** |
| --- | --- | --- | --- |
| Ni_4.5_Fe_4.5_S_8_ | 73-0515 | NiS | 89-1956 |
| CuFeS_2_ | 83-0983 | CuSO_4_ | 72-0090 |
| Fe_7_S_8_ | 89-1954 | Fe_2_(SO_4_)_3_ | 73-0148 |
| Fe_3_O_4_ | 99-0073 | Fe_2_O_3_ | 89-0597 |
| FeS_2_ | 71-1680 | NiO | 89-7130 |
| SiO_2_ | 85-0865 | NiSO_4_ | 13-0435 |
| Mg_3_Si_2_O_5_(OH)_4_ | 86-0404 | Cu_0.67_Fe_2.33_O_4_ | 73-2317 |
| Fe_2_O_3.5_Fe_2_(SO_4_)_3_ | 16-0896 | Ni_0.4_Fe_2.6_O_4_ | 87-2336 |
| (Mg,Fe)SiO_3_ | 74-1393 | CuO.CuSO_4_ | 46-0005 |
| (Mg,Fe)_2_Si_2_O_6_ | 88-1914 | Cu_2_S | 83-1462 |
| Ni_2_SiO_4_ | 80-0940 | MgSO_4_ | 72-1259 |
